# Mexican Households’ Purchases of Foods and Beverages Vary by Store-Type, Taxation Status, and SES

**DOI:** 10.3390/nu10081044

**Published:** 2018-08-08

**Authors:** Lilia S. Pedraza, Barry M. Popkin, Juan C. Salgado, Lindsey S. Taillie

**Affiliations:** 1 Department of Nutrition, University of North Carolina, Chapel Hill, NC 27599-7461, USA; lpedraza@email.unc.edu (L.S.P.); popkin@unc.edu (B.M.P.); 2 Department of Health Policy and Management, University of North Carolina, Chapel Hill, NC 27599-7411, USA; jsalgado@unc.edu

**Keywords:** food purchases, retailers, socioeconomic factors, taxes, Mexico

## Abstract

Where people shop for food is often linked to the healthiness of food purchases. In Mexico, no research has examined the connection between where people shop, what they buy, and their socioeconomic status (SES). Mexico’s sugary beverage and junk food taxes have made households decrease purchases of taxed products. However, whether households have changed where they shop is unknown. To address this gap, we use a repeated cross-sectional analysis of household packaged food and beverage purchases from the Nielsen Mexico Consumer Panel Survey from 2012 to 2015 (*n* > 5500 households). We examine changes in the volume of the purchase of taxed and untaxed products from different store-types (i.e., convenience stores, supermarkets, traditional retailers, wholesalers, home water-delivery, and others) by SES using multivariate linear regression models. Results show that high-SES households purchased more foods and beverages at all store-types except for low-SES who purchased the most foods and taxed beverages at traditional retailers. Purchases of taxed foods and beverages from traditional retailers significantly decreased for low-SES and middle-SES households and from supermarkets for middle-SES and high-SES households. Purchases of untaxed beverages from wholesalers significantly increased for middle-SES households and from convenience stores for high-SES households. Our findings suggest that consumers choose different stores to purchase beverages than to purchase foods and that taxes may have differentially affected each store-type.

## 1. Introduction

Diet quality is a leading contributor to obesity, diabetes, and other non-communicable diseases (NCDs) [1]. Where people shop for food could influence the healthiness of what they buy and subsequently consume. In high-income countries, there is growing research linking the food environment including the type of stores (e.g., convenience stores and supermarkets) and the availability of products to the shopping behavior, food choices, and diet of consumers [2,3,4,5,6]. Evidence from the US suggest, that relative to other food stores, supermarkets tend to offer more variety of high-quality foods at lower cost than convenience stores, which tend to offer high-calorie foods at higher prices [3,5,7]. Similarly, in high-income-countries, access to and the purchase of different foods is influenced by individuals’ socioeconomic status (SES) [8]. For example, in the US, rural, low-income, and minority populations have less access to supermarkets and to healthy foods than their urban and higher-income counterparts [3,9]. However, the majority of research to date has focused on high-income countries and not much is known about whether the nutritional quality of food and beverage purchases differs by store type in low-and-middle income countries (LMICs). Recent evidence suggests that foods purchased at supermarkets in LMICs are in general more processed than foods purchased at smaller self-service stores [10,11,12]. However, there has been very little research examining the differences in food purchasing behaviors by socioeconomic indicators in LMICs [13,14]. Food purchasing choices are related to the availability of these products at home [4]. Food availability in the households influence individual dietary intake and ultimately weight status. Identifying potential differences in food shopping by SES is additionally important in LMICs because evidence has shown that, even though the prevalence of overweight and obesity in most LMICs is higher among the highest SES groups as measured by the wealth quintile and the education group, some LMICs have shown a faster growth rate in the overweight in the lowest SES groups [15]. These findings imply that the risk factors and burden of chronic diseases are not equally distributed across SES groups in LMICs. 

Mexico is a useful setting in which to explore the association between the store type and the nutritional quality of food and beverage purchases in LMICs. In Mexico, the entry to the market of multinational chains drove a rapid rise and spread of supermarkets and modern convenience stores in the early 1990s with food retailing shares growing from 10% to 20% in 1990 to supermarkets accounting for 68% of overall food grocery sales (including hot, soft, and alcoholic drinks and fresh and packaged foods) by 2016 [16,17,18,19]. Supermarket consolidation substantially displaced traditional retailing and fresh markets. However, traditional food outlets remain of high cultural importance in urban and rural Mexican localities and, in 2016, held 32% of the overall food sales [17,18]. The nationally representative Mexican Health and Nutrition Survey of 2012 (Encuesta Nacional de Salud y Nutrición, ENSANUT 2012) found that 58% of food calories came from packaged, processed foods [20]. Moreover, according to the Pan American Health Organization, Mexico is the second largest per-capita consumer of energy-dense, ultra-processed food and drinks in Latin America [21] including sugar-sweetened beverages and foods high in saturated fat or sugar, which combined contributed to 26% of the total energy intake of the Mexican population in 2012 [22]. Concurrently, excess weight in the Mexican population increased rapidly over the past several decades and is currently among the highest in the world with 73% of adults, 36% of adolescents, and 33% of children overweight or obese [23]. As suggested by evidence from other countries, food retailers may play an important role in Mexico’s current obesity epidemic, but, to date, there is no clear understanding of the type of stores where households with different socioeconomic characteristics shop for food and what types of foods they purchase.

Several strategies have been implemented to prevent a further rise in the prevalence of overweight and diabetes in Mexico including fiscal measures such as a one peso per liter (≈10%) tax on sugar-sweetened beverages (SSBs) and an 8% tax on non-essential energy dense (>275 kcal/100 g) foods [24,25,26] that were enforced at the national level in January of 2014. These taxes are collected directly from the products manufacturers regardless of where products are sold [27,28]. The sustained average decreases in the purchases of packaged foods and beverages subject to taxation of 8% and 6%, respectively, were observed in the first two years after taxation [29,30]. However, where households purchase the taxed and untaxed products remains unexplored.

This study helps to fill this gap in the literature by describing the proportion of the total volume of the taxed and untaxed packaged foods and beverages purchased in Mexico at each store-type and socioeconomic level using detailed food purchase data from a nationally representative sample of Mexican urban households from 2012 to 2015. It also compares whether the proportion of purchases of products by taxation status and store-type significantly differed between 2012 and 2015. Understanding the role of store-types in food and beverage purchase choices could help develop interventions that influence the food environment and in-store purchasing decisions to promote healthier eating practices in Mexico.

## 2. Materials and Methods 

### 2.1. Dataset

This study used volume information of packaged food and beverage household purchases from the Nielsen Company’s Mexico Consumer Panel Services (CPS) 2012 to 2015 that is equivalent to the data from the US Nielsen Homescan panel [31]. Nielsen CPS randomly samples households from 53 cities with more than 50,000 inhabitants (including Mexico City) in 28 states in Mexico and contacts them for recruitment through postcards and letters [32]. Nielsen CPS weights each household according to their composition, locality, and socioeconomic measures through iterative proportional fitting that matches demographic estimates from the National Institute of Statistics and Geography (Instituto Nacional de Estadística Geografía e Informática, INEGI). The household weights provided by Nielsen CPS vary by year to ensure the representativeness of the Mexican urban population. There is a 6% to 8% annual sample rotation of households coming in and out of the sample and when households drop out of the panel, new households are selected from the sample frame for that year. Nielsen CPS has been previously used in several studies to evaluate the effect of the taxes on sugary beverages and non-essential energy dense foods on purchases in Mexico [29,30,33,34].

Nielsen CPS collects information only of packaged products with an available barcode, which lacks purchasing data for non-packaged foods such as produce, meats, prepared foods, and other random weight products. However, package produce like canned fruits are included. 

Households keep shopping receipts, empty product packages, and purchasing diaries. A trained interviewer visits the household every two weeks to collect the information, check the pantry, and re-scan all available items with a barcode. The purchase place, date, price paid, and number of units are recorded for each purchase. This study included all 6645 available households from 2012–2015, which translated into 263,145 household-year observations. From the overall household sample, 4726 unique households consistently appeared every year from 2012 to 2015. 

Stores were categorized into the following groups: convenience stores, supermarkets, wholesalers, traditional retailers (usually attended by the owner includes traditionally fixed stores installed in permanent public markets), and others (e.g., department stores, pharmacies, movie theaters, etc.) (Supplemental Table S1) [35,36]. An additional home water-delivery category was included for beverages only since the routine purchase and home-delivery of 20 liter-jugs of potable water is a common practice in Mexican households. 

Trained dietitians classified 302,788 unique barcodes from households’ food and beverage purchases into the relevant food group and taxation status according to the Mexican law mandate [26]. Since products within one food and beverage group can have different characteristics under the Mexican legislation, there are cases where different food items within a food group can be taxed or untaxed. For beverages, the taxation status depended on whether sugar was added or not (e.g., sodas with added sugar versus sodas with non-caloric sweeteners) while the food and taxation status was assigned depending on whether they reached the energy density cutoff value for taxation (275 kcal/100 g). For example, salty snacks with ≥275 kcal/100 g are classified as taxed while salty snacks with <275 kcal/100 g are classified as untaxed. On the contrary, if a food does not reach the legislation’s cut-off value, like in the case of canned fruits or fruit-based sweets, then that group will only appear in the untaxed category. Foods and beverages considered essential or staples such as tortillas and milk are always excluded from taxation and appear only in the untaxed categories. Food categories that were not collected during the four years like tortillas, bread from a bakery, chocolates, and candies were excluded from the analyses. Details on food and taxation categorization are presented in Supplemental Table S2. 

This study used the socioeconomic (SES) categories (low, medium, and high) provided by Nielsen CPS that are defined based on an eight-category measure derived from annually updated questions on household assets (i.e., number of bathrooms, number of bedrooms, number of automobiles owned, shower, gas stove, number of light bulbs, and type of floor) and educational attainment of the head of the household. Nielsen CPS has validated this measure of SES and updates it annually with new data from each sample household. These SES constructs have been previously used in research on food and beverage purchases in Mexico [30,33,34]. 

### 2.2. Statistical Analyses

A repeated cross-sectional analysis from 2012 to 2015 was conducted to assess the changes over time in each store-type contributions to the volume of foods and beverage purchases. The contribution of each store-type was calculated as a percentage of the total purchase volume of foods and beverages and the percentage of taxed/untaxed product purchases. The per capita per day purchases of foods and beverages were calculated by dividing the total annual volume of purchases by household size by 365. First, we examined whether there was a difference in the volume of taxed and untaxed food and beverage purchases by store type by SES. Second, we examined whether there were differential changes by SES over time. Households with no purchases of taxed/untaxed products were included in the analysis with a percentage contribution or per capita purchase of zero. Changes over time in the contribution to purchases and total daily per capita volume of purchases by store-type and SES were examined using multivariate linear regressions by considering a *p* < 0.05 as statistically significant. All analyses were conducted using STATA version 14 (StataCorp, College Station, TX, USA) and were adjusted using Nielsen household projection factors. 

## 3. Results

From 2012 to 2015, more than 50% of the Nielsen Company’s Mexico Consumer Panel Services (CPS) households belonged to the middle socioeconomic strata (SES). Virtually all (>99%) of households purchased at least one item of food or beverages from traditional retailers and 99% from supermarkets in any given year. Roughly 75% of households shopped for foods and beverages at convenience stores, 38% of households purchased foods and beverages at wholesalers, and nearly 90% of households purchased some foods or beverages in other types of stores such as pharmacies, movie theaters, department stores, and more. A total of 80% of the households purchased 20 liter-jugs of potable water that were delivered to their homes (Supplemental Table S3). 

### 3.1. Percentage of Beverage and Food Purchases by Store-Type

In 2015, 37% of total beverage purchases were received via home water-delivery, 35% of total beverage purchases were made in traditional retailers, 12% in other retailers, 11% in supermarkets, and 5% in convenience stores. From 2012 to 2015, small but statistically significant changes were observed in the percentage of total beverages purchased in convenience stores, which increased one percentage point (*p*-value < 0.05). During the same time period, the amount of beverages purchased from traditional retailers decreased two percentage points (*p*-value < 0.01) (Table S1). In low-SES households, beverage purchases from traditional retailers decreased from 46% to 41% (*p*-value < 0.01) while this was not the case in other SES households. In the same period, high-SES households increased their purchases of beverages in convenience stores from 4% to 6% (*p*-value < 0.05) (Table 1). 

Overall, 73% of taxed beverages were purchased from traditional retailers while 19% were purchased in supermarkets and 3% in convenience stores in 2015. From 2012 to 2015, middle and high-SES households increased their purchases of taxed beverages from convenience stores but decreased it in supermarkets (*p*-value < 0.05). High-SES households decreased their purchases of taxed beverages at supermarkets as well (*p*-value < 0.01). In 2015, 43% of the purchases of untaxed beverages occurred through home water-delivery followed by 25% from traditional retailers, 12% from supermarkets, and 6% from convenience stores. Untaxed beverages purchased showed no significant changes by store-type and SES from 2012 to 2015. See Supplemental Table S3 for all mean percentage purchases of taxed and untaxed beverages by SES at all points in time.

In 2015, 49% and 41% of total foods were purchased at supermarkets and traditional retailers, respectively. From 2012 to 2015, total food purchases significantly increased at wholesalers but decreased at traditional retailers and other retailers (*p*-value < 0.05). Low-SES households increased their food purchases in supermarkets and decreased them at traditional retailers while middle-SES households increased their food purchases at wholesalers but decreased them at traditional retailers (*p*-value < 0.05). High-SES households show no significant change in food purchases in any store-type (Table 1). The same trends in taxed food purchases from different store-types over time were observed by SES. See Supplemental Table S4 for all mean percentage purchases of taxed and untaxed foods by SES at all points in time.

### 3.2. Differences in Volume Purchases of Beverages by Taxation Status and SES from 2012 to 2015

From 2012 to 2015, purchases of taxed beverages declined by 12 mL/capita/day (−34%), 2 mL/capita/day (−56%) and 32 mL/capita/day (−18%) in supermarkets, wholesalers, and traditional retailers, respectively (relative percentage changes are presented in parenthesis). Purchases of untaxed beverages by all households in the sample increased by 5.3 mL/capita/day (11%) and 1.9 mL/capita/day (46%) (*p*-value < 0.05) at supermarkets and wholesalers, respectively. See Supplemental Table S5 for all mean daily per capita purchases of total, taxed, and untaxed beverages by SES during all years.

Purchases of taxed and untaxed beverages differed across SES since 2012 to 2015 changes were mainly observed among traditional retailers in the low-SES households while changes were concentrated in supermarkets in the middle-SES and high-SES households. Purchases of taxed beverages decreased by 45 mL/capita/day (22%) at traditional retailers in the low-SES households, by 36 mL/capita/day (−19%) and 11 mL/capita/day (−31%) at traditional retailers and supermarkets in the middle-SES households, and by 23 mL/capita/day (−41%), 8 mL/capita/day (−69%), and 3 mL/capita/day (−49%) in supermarkets, wholesalers, and other retailers, respectively, in high-SES households (*p*-value < 0.01). Purchases of untaxed beverages showed a significant increase from 2012 to 2015 of 1.8 mL/capita/day (90%) at wholesalers in the middle-SES households and purchases increased by 18 mL/capita/day (60%) at convenience stores in the high SES households (*p*-value < 0.05) (Supplemental Table S5).

**Figure 1:**
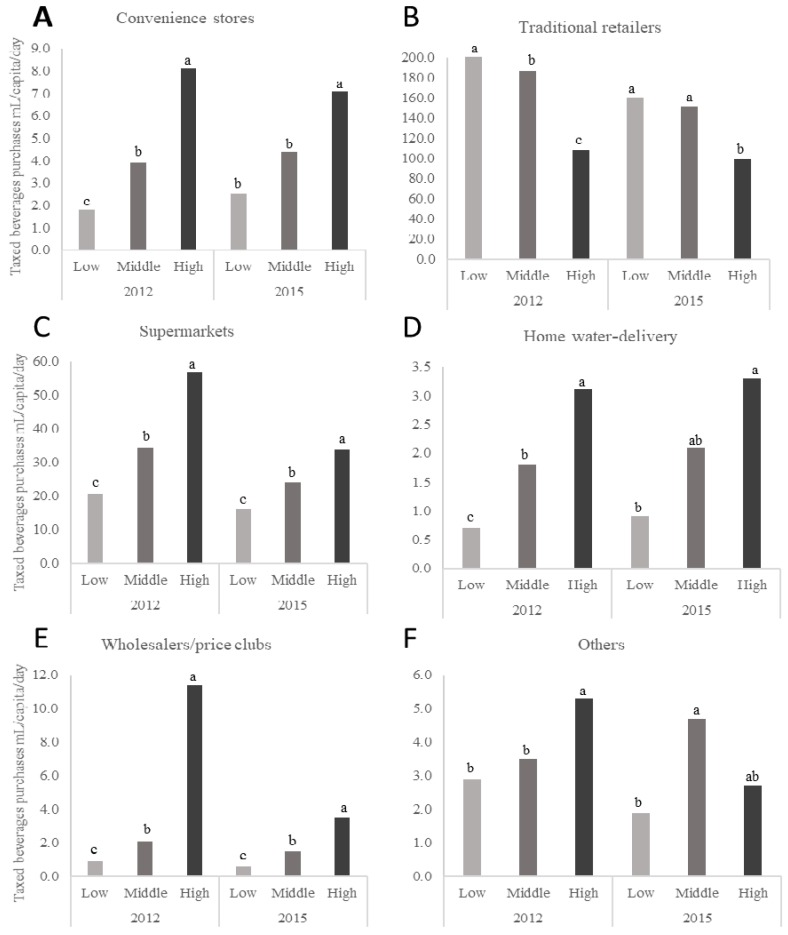
Daily per capita purchases of taxed beverages in urban Mexican households, according to socioeconomic status (SES), for the years 2012 and 2015. *n* = 959 in the low-SES, *n* = 3133 in the medium-SES, and *n* = 1721 in the high-SES group in 2012 and *n* = 1170 in the low-SES, *n* = 2690 in the medium-SES, and *n* = 1633 in the high-SES group in 2015. Subgraphs refer to taxed beverage purchases by store-type including (**A**) convenience stores, (**B**) traditional retailers, (**C**) supermarkets, (**D**) home water-delivery, (**E**) wholesalers and price clubs, and (**F**) Other sources. Values represent weighted unadjusted means. SES classification is based on the socioeconomic index provided by Nielsen. Multivariate linear regression models were used to predict per capita daily purchases of taxed beverages according to store-type, SES, and year of purchases. Data presented are restricted to the Nielsen Company’s Mexico Consumer Panel Services purchasing information for 2012 and 2015 only for clarity. However, *p*-values represent the *p*-trend for years 2012, 2013, 2014, and 2015. Labeled means with a different letter represent significant differences between the SES categories means (*p*-trend < 0.05) where the letters ^a,b,c^ are used to differentiate the highest, middle, and lowest mean values for each figure in the panel. Source: Authors’ own analyses and calculations based on data from Nielsen through its Mexico Consumer Panel Service for the food and beverage categories for January 2012 to December 2015. The Nielsen Company, 2016. Nielsen is not responsible for and had no role in preparing the results reported herein.

**Figure 2:**
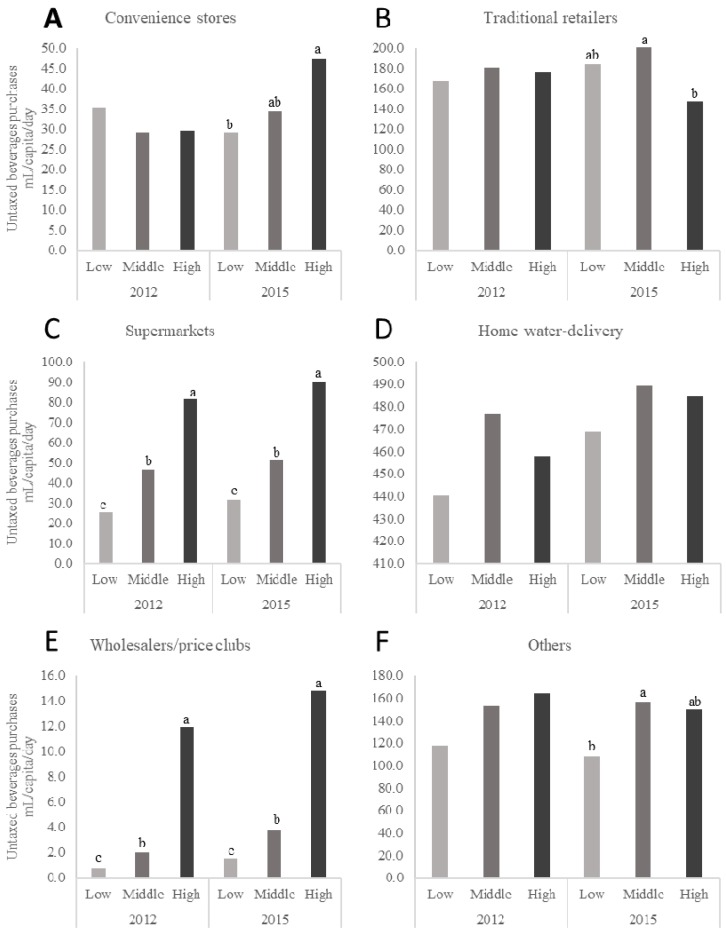
Daily per capita purchases of untaxed beverages in urban Mexican households according to SES for the years 2012 and 2015. *n* = 959 in the low-SES, *n* = 3133 in the medium-SES, and *n* = 1721 in the high-SES group in 2012 and *n* = 1170 in the low-SES, *n* = 2690 in the medium-SES, and *n* = 1633 in the high-SES group in 2015. Subgraphs refer to untaxed beverage purchases by store-type including (**A**) convenience stores, (**B**) traditional retailers, (**C**) supermarkets, (**D**) home water-delivery, (**E**) wholesalers and price clubs, and (**F**) Other sources Values represent weighted unadjusted means. SES classification is based on the socioeconomic index provided by Nielsen. Multivariate linear regression models were used to predict per capita daily purchases of untaxed beverages according to store-type, SES, and year of purchases. Data presented are restricted to the Nielsen CPS purchasing information for 2012 and 2015 only for clarity. However, *p*-values represent the *p*-trend for years 2012, 2013, 2014, and 2015. Labeled means with a different letter represent significant differences between the SES categories means (*p*-trend < 0.05) where the letters ^a,b,c^ are used to differentiate the highest, middle, and lowest mean values for each figure in the panel. Source: Authors’ own analyses and calculations based on data from Nielsen through its Mexico Consumer Panel Service (CPS) for the food and beverage categories for January 2012 to December 2015. The Nielsen Company, 2016. Nielsen is not responsible for and had no role in preparing the results reported herein.

The daily per capita purchases of taxed beverages by store type and SES for 2012 and 2015 are presented in Figure 1 while daily per capita purchases of untaxed beverages for the same time points and SES are presented in Figure 2. Figure 1 and Figure 2 only present mean values for the years 2012 and 2015 but represent the changes across the 2012 through 2015 study period with a *p*-trend. The high-SES households purchased more volume of taxed beverages at any given store-type except for purchases of taxed beverages from traditional retailers where the low-SES households purchased more than the high-SES households (206 vs. 109 mL/capita/day) in 2012 and (161 vs. 99 mL/capita/day) in 2015. The middle-SES households purchased more volume of untaxed beverages at any given store-type than the low-SES and high-SES households did. 

### 3.3. Differences in Volume Purchases of Foods by the Taxation Status and SES from 2012 to 2015

From 2012 to 2015, purchases of taxed foods significantly decreased by 1 g/capita/day (−18%), 2 g/capita/day (−30%), and 0.3 g/capita/day (−40%) from supermarkets, traditional retailers, and other retailers respectively (*p* < 0.001) while untaxed foods purchases decreased by 2 g/capita/day (−8%), 3 g/capita/day (−15%), and 1 g/capita/day (−29%) g per capita daily from supermarkets, traditional retailers, and other retailers (*p* < 0.05). See Supplemental Table S6 for all mean daily per capita purchases of total, taxed, and untaxed foods by SES at all years.

Purchases of taxed and untaxed foods from supermarkets, traditional stores, and other retailers decreased from 2012 to 2015 in all SES households. Purchases of taxed foods decreased over time by 3 g/capita/day (−38%) and 0.3 g/capita/day (−60%) at traditional retailers and other retailers, respectively, in low−SES households. Taxed foods purchases decreased by 2 g/capita/day (−28%) and 1 g/capita/day (−17%) at traditional retailers and supermarkets and increased by 0.2 g/capita/day (40%) at wholesalers in middle-SES households. Purchases of taxed foods decreased by 3 g/capita/day (−25%), 1 g/capita/day (−16%), and 0.3 g/capita/day (−43%) from supermarkets, traditional stores, and other retailers in the high-SES households (*p* < 0.05). Similarly, purchases of untaxed foods decreased by 4 g/capita/day (−18%) and 1 g/capita/day (−35%) from traditional stores and other retailers in the low-SES households, decreased by 3 g/capita/day (−16%) and 1 g/capita/day (−27%) from traditional retailers and other retailers in the middle SES households, and decreased by 6 g/capita/day (−16%) and 1 g/capita/day (−25%) from supermarkets and traditional retailers in high SES households (*p* < 0.05) (Supplemental Table S6).

**Figure 3:**
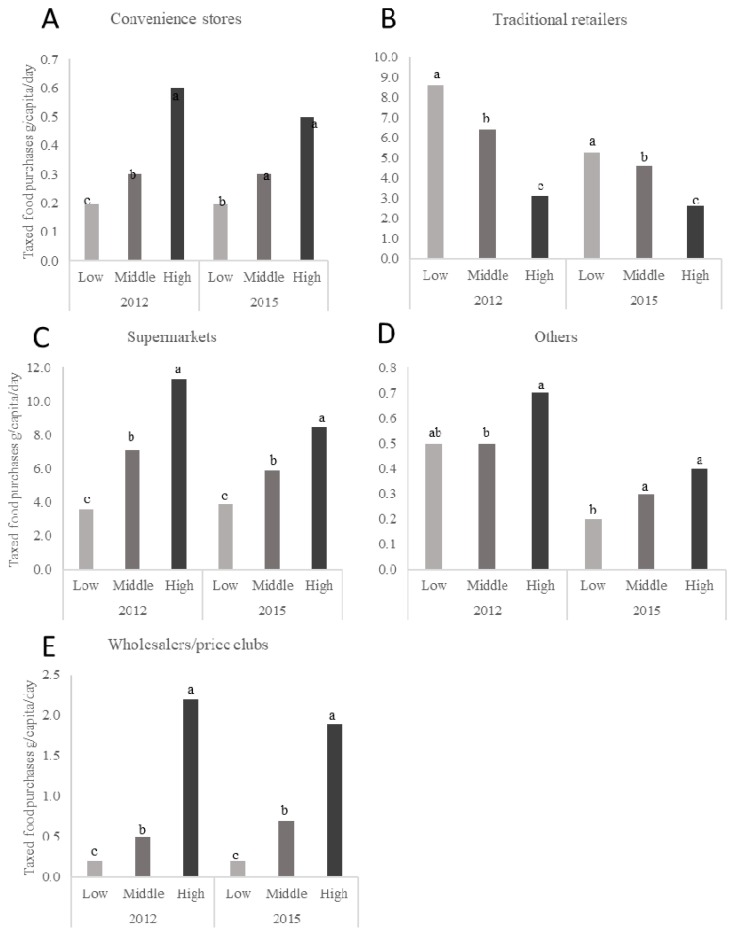
Daily per capita purchases of taxed foods in urban Mexican households, according to SES, for the years 2012 and 2015. *n* = 959 in the low-SES, *n* = 3133 in the medium-SES, and *n* = 1721 in the high-SES group in 2012 and *n* = 1170 in the low-SES, *n* = 2690 in the medium-SES, and *n* = 1633 in the high-SES group in 2015. Subgraphs refer to taxed food purchases by store-type including (**A**) convenience stores, (**B**) traditional retailers, (**C**) supermarkets, (**D**) home water-delivery, and (**E**) wholesalers and price clubs. Values represent weighted unadjusted means. SES classification is based on the socioeconomic index provided by Nielsen. Multivariate linear regression models were used to predict per capita daily purchases of taxed foods according to store-type, SES, and year of purchases. Data presented are restricted to the Nielsen CPS purchasing information for 2012 and 2015 only for clarity. However, *p*-values represent the *p*-trend for years 2012, 2013, 2014, and 2015. Labeled means with a different letter represent significant differences between the SES categories means (*p*-trend < 0.05) where the letters ^a,b,c^ are used to differentiate the highest, middle, and lowest mean values for each figure in the panel. Source: Authors’ own analyses and calculations based on data from Nielsen through its Mexico Consumer Panel Service (CPS) for the food and beverage categories for January 2012 to December 2015. The Nielsen Company, 2016. Nielsen is not responsible for and had no role in preparing the results reported in this study.

**Figure 4:**
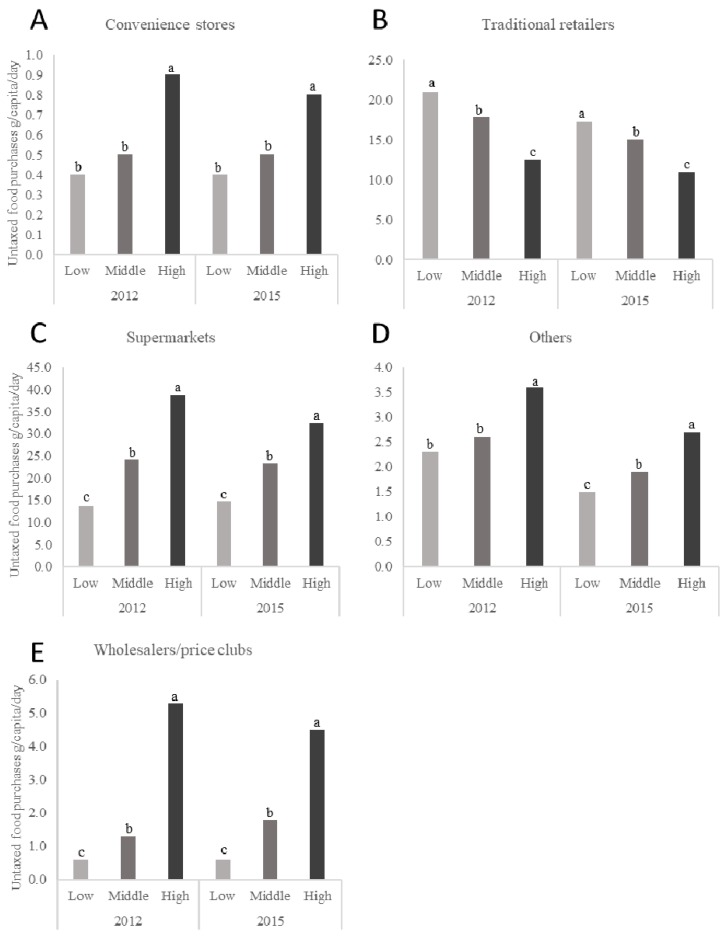
Daily per capita purchases of untaxed foods in urban Mexican households, according to SES, for the years 2012 and 2015. *n* = 959 in the low-SES, *n* = 3133 in the medium-SES, and *n* = 1721 in the high-SES group in 2012 and *n* = 1170 in the low-SEs, *n* = 2690 in the medium-SES, and *n* = 1633 in the high-SES group in 2015. Subgraphs refer to untaxed food purchases by store-type including (**A**) convenience stores, (**B**) traditional retailers, (**C**) supermarkets, (**D**) home water-delivery, and (**E**) wholesalers and price clubs. Values represent weighted unadjusted means. SES classification is based on the socioeconomic index provided by Nielsen. Multivariate linear regression models were used to predict per capita daily purchases of untaxed foods according to store-type, SES, and year of purchases. Data presented are restricted to the Nielsen CPS purchasing information for 2012 and 2015 only for clarity. However, *p*-values represent the *p*-trend for years 2012, 2013, 2014, and 2015. Labeled means with a different letter represent significant differences between the SES categories means (*p*-trend < 0.05) where the letters ^a,b,c^ are used to differentiate the highest, middle, and lowest mean values for each figure in the panel. Source: Authors’ own analyses and calculations based on data from Nielsen through its Mexico Consumer Panel Service (CPS) for the food and beverage categories for January 2012 to December 2015. The Nielsen Company, 2016. Nielsen is not responsible for and had no role in preparing the results reported in this paper.

The daily per capita purchases of taxed foods by store type and SES for 2012 and 2015 are presented in Figure 3 while daily per capita purchases of untaxed foods for the same years are presented in Figure 4. Figure 3 and Figure 4 only present mean values for the years 2012 and 2015, but represent the changes across the 2012 through 2015 study period with a *p*-trend. As observed in beverages, the high-SES households purchased a higher volume of taxed and untaxed foods in general than the other SES households except for purchases of any foods, taxed and untaxed, from traditional retailers where the low-SES households purchased significantly more than the other SES households.

## 4. Discussion

To our knowledge, this is the first study to report the absolute and relative purchases of foods and beverages by store-type and taxation status in the Mexican population. Regardless of the year and excluding the home water-delivery category since it mostly represents water purchases, the highest volume of purchases of taxed and untaxed beverages was observed at traditional retailers across all SES subpopulations. Conversely, the highest volume of both taxed and untaxed food purchases by the total population was observed in supermarkets except among the low-SES households where purchases of all foods were highest at traditional retailers. 

These results are mixed when compared to findings on purchases of packaged products by store-type in the US. Stern et al. [37] reported that grocery-chains, which is a store category comparable to the supermarket category in this analysis, were the biggest contributor to total volume of household purchases. However, the differences between studies might be explained by our analysis separating food purchases from beverage purchases while Stern reported them combined by differences in consumer behavior between countries or by differences in the store availability between Mexico and the US. 

Our analyses show that Mexican households choose different stores to purchase foods than to purchase beverages. While the reason for choosing different store-types to buy different products remains unclear, one possible explanation for the overall beverage purchasing pattern we observed could be the recently described growing preference of the Mexican population towards doing more regular shopping trips in smaller stores such as traditional retailers and convenience stores rather than large shopping trips in large stores such as supermarkets [18]. Another possibility is that, contrary to food purchasing and beverage purchasing excluding potable water could be more likely to be the result of unplanned or impulse buying. This effect may have increased over time due to the large and increasing density of traditional retailers available across cities. Nonetheless, Nielsen CPS does not provide information on store density data to confirm this hypothesis. 

We also observed several differences by household SES. First, this study shows that low-SES households rely on traditional retailers to obtain most of their foods and beverages by purchasing more overall products in traditional retailers than the other SES households. In contrast, the middle and upper SES households purchased foods mainly from supermarkets and beverages mostly from traditional retailers. Some possible explanations for the observed difference in food sources from different SES households could be related to low-SES households visiting supermarkets only once a week to do a larger purchase of products while visiting traditional stores three times a week or more to do the shopping needed for the moment (e.g., milk, bread, or soda) [38]. Additionally, low-SES households might prefer fresh produce, which is usually cheaper, over the packaged or frozen options offered at supermarkets that have become more convenient for the medium-SES and high-SES households facing longer working hours. Another contributing factor to the higher purchases in supermarkets from the middle and high-SES could be the extended hours of service offered by supermarkets compared to traditional retailers that allows working consumers to do their shopping after working hours. Transportation might also play a big role in the food source that a household chooses since consumers without access to a car or other convenient transportation method could choose to buy smaller amounts at closer sources (e.g., traditional stores) to be able to carry back their purchases as opposed to the larger amounts of shopping and traveling distance that having a car permits [38]. Pricing and product variety and availability could also influence a household’s preference for a particular store-type [39]. Supermarkets might have a larger array of products to choose from but these could be pricier than at traditional retailers. However, middle and high SES households might be willing to pay more to gain the convenience from shopping at supermarkets [35,39]. It remains important to explore the differences in product availability at different store-types since it could influence the nutrient quality of the products that the households are purchasing and likely their dietary intake. Differences in the nutrient quality of products at different store-types could reinforce dietary SES inequalities in the Mexican population. 

In our study, the higher-SES households purchased relatively more taxed foods and untaxed beverages and foods than the lower-SES households while the opposite was observed for taxed beverages. Considering the taxation status of the foods and beverages analyzed in this paper as proxy for the healthiness of the products, the observed socioeconomic disparities of purchases of healthier and unhealthier products are consistent with those observed by López-Olmedo et al. [40] using information from the Mexican National Health and Nutrition Survey 2012 and data from the Nielsen Mexico Consumer Panel Service Dataset 2012–2014.

We also observed variation in differences in the purchases of taxed foods and beverages by SES with low-SES households showing larger declines. These findings were expected given previous findings from the evaluations of the Mexican taxation on sugar-sweetened beverages and high-energy non-basic foods where the lower SES-households have shown the greatest reduction in the per capita volume of taxed food and beverage purchases compared to middle and high-SES households [29,30,33,34]. However, we obtained novel results by showing that purchases of taxed foods and beverages decreased at traditional retailers only in the low and middle SES while taxed foods purchases decreased in supermarkets only in the middle and high SES households. These significantly different purchasing patterns of taxed and untaxed products from different types of stores and SES could reflect underlying aspects of store choice including physical proximity, price, promotions, and preferences. Since store choice has been associated with diet quality and health [41], it remains important to explore what factors influence store choice and purchasing patterns in the Mexican population. 

### Limitations

The key limitation in our study is that the data collected by Nielsen CPS contains information only of packaged products and lacks purchasing data for fruits, vegetables, and other products sold in bulk, which provides a partial picture of Mexican household’s food and beverage purchases. Moreover, given the nature of the data collection, we lack information of food outlets that offer mostly fresh produce such as wet and open markets. However, according to data from the 2016 National Household Income and Expenditure Survey (ENIGH) [42], 13% and 8% of all purchases are made in markets in rural and urban areas, respectively, where almost the totality of purchases are fresh produce. Therefore, our analyses of packaged foods and beverage purchases are not likely to be affected. Furthermore, even though participants are instructed to keep all food and beverages packages, there is the potential for individuals to fail to keep packages that are purchased “on the go.” Therefore, the under-reporting of products mainly consumed away from home is a potential limitation. 

Another limitation of our research is that our data are representative only of the urban regions of the country. Rural households could also present a different purchasing behavior due to a variety of store-types in these areas as opposed to urban settings. However, statistics from the National Institute of Statistics and Geography (Instituto Nacional de Estadística Geografía e Informática, INEGI) indicate that the Nielsen CPS data represented 63% of the Mexican population and 75% of foods and beverage expenditures in 2014 [33]. Furthermore, previous studies using ENIGH information found that, after taxation, the magnitude of decrease in SSBs purchases was greater in urban households than in rural households. This is likely because, in less-populated rural and small town areas, after the tax was passed, prices were increased less [43,44]. Therefore, our results provide relevant evidence for a significant part of the Mexican population where the fiscal regulation has shown a stronger effect on the prices of the targeted products.

An additional limitation is our lack of time-varying nutritional data for the 2012 to 2015 period covered in this study during our data processing (2014–2015). All Nielsen CPS 2012–2015 beverages and foods were assigned a taxation status based on the nutritional information that the products and some retailers had available on their websites at that time. Therefore, all data was categorized based on their post-tax values and tax status should have remained consistent over time. However, manufacturers might have reformulated their products to achieve an energy density below the legal threshold before or around the time that the tax was enforced. We were unable to examine changes in reformulation in this study due to our shortage of nutritional information. Nonetheless, future research should examine whether reformulation occurred and how much of the observed changes in purchases were due to product reformulation versus changes in consumer behavior. 

An important consideration for this study is the potential for some of the observed changes in the purchases of packaged products over time to be statistically significant due to the large size of the database and not because of the magnitude of the changes relevant for a particular store-type. Further research is needed to evaluate whether these small but significant changes are reflected in other characteristics of the purchased products such as their nutrient profile.

It is also important to emphasize that the purpose of this study was to describe how much of the taxed and untaxed packaged products were being purchased at each store-type without trying to identify the determinants of the observed differences. Therefore, other potential explanations for the observed results such as the influence on purchasing behavior of the types of products offered at each store-type before and after the taxation enforcement should be subject to further analysis. 

Future studies focusing on characterizing the motivation of consumers to shop at a certain store-type and certain products should consider the potential for selectivity due to customer shopping motivations or to store-specific factors.

## 5. Conclusions

This study is the first based on our knowledge to study packaged foods and beverage purchasing at different store-types and different SES in the Mexican population. Our findings suggest that different stores are chosen to purchase beverages than to purchase foods. Though most taxed and untaxed beverages were purchased at traditional retailers, the store-types where households obtained their food varied across SES since low SES households primarily visited traditional retailers and the middle and high SES households visited supermarkets. Results from this study suggest existing underlying factors influencing differentially purchasing behavior and store choice by SES households and might indicate that, despite overall lack of an employment effect from the two Mexican sugary beverage and nonessential food taxes, each store-type were affected differentially [45]. Further research is needed to provide evidence of the association between store choice, purchasing behavior, and diet quality in Mexico.

## Figures and Tables

**Table 1:** Unadjusted percentage mean of total beverage and food purchases from 2012 to 2015 by store-type and socioeconomic status.

	Total Population					Low SES					Medium SES						High SES			
	2012	2013	2014	2015	*p* for trend	2012	2013	2014	2015	*p* for trend	2012	2013	2014	2015	*p* for trend	2012	2013	2014	2015	*p* for trend
	*n* = 5813	*n* = 5775	*n* = 5657	*n* = 5493		*n* = 959	*n* = 1094	*n* = 1087	*n* = 1170		*n* = 3133	*n* = 2872	*n* =2 815	*n* = 2690		*n* = 1721	*n* = 1809	*n* = 1755	*n* = 1633	
**Total beverage purchases (%±SE)**																				
Convenience stores																				
	4.0	4.5	4.7	4.8	0.019	4.4	4.8	4.7	4.2	0.615	3.8	3.8	4.2	4.4	0.098	4.2	5.4	5.7	6.1	0.025
	± 0.2	± 0.2	± 0.2	± 0.3		± 0.4	± 0.4	± 0.4	± 0.5		± 0.2	± 0.2	± 0.3	± 0.4		± 0.4	± 0.5	± 0.6	± 0.7	
Supermarkets	11.3	11.6	11.2	10.8	0.191	6.6	7.7	7.3	7.5	0.411	10.7	11.3	11.1	10.1	0.325	17.4	16.2	15.6	16	0.221
	± 0.3	± 0.3	± 0.3	± 0.4		± 0.5	± 0.6	± 0.6	± 0.7		± 0.4	± 0.5	± 0.5	± 0.5		± 0.7	± 0.7	± 0.7	± 1	
Wholesalers/price clubs	0.9	0.9	1.0	1.1	0.181	0.2	0.3	0.3	0.2	0.684	0.5	0.6	0.8	0.7	0.042	2.5	2.4	2.1	2.7	0.808
	± 0.1	± 0.1	± 0.1	± 0.1		± 0	± 0.1	± 0.1	± 0		± 0.1	± 0.1	± 0.1	± 0.1		± 0.2	± 0.2	± 0.2	± 0.5	
Traditional retailers	37	36.4	35.8	34.7	0.006	46	43.6	42.4	40.7	0.006	37.9	38	37.1	36.4	0.161	26.2	26	25.6	24.1	0.199
	± 0.6	± 0.6	± 0.6	± 0.7		± 1.3	± 1.3	± 1.4	± 1.4		± 0.7	± 0.8	± 0.9	± 1		± 1.1	± 1.1	± 1.1	± 1.2	
Home water-delivery	34.8	34.9	34.9	36.8	0.054	33.5	33.7	34.9	37.2	0.054	34.7	33.9	33.6	35.6	0.625	36.1	38.1	37.9	39.2	0.197
	± 0.6	± 0.7	± 0.7	± 0.8		± 1.3	± 1.4	± 1.5	± 1.6		± 0.8	± 0.9	± 1	± 1.2		± 1.3	± 1.4	± 1.5	± 1.7	
Others	12.0	11.7	12.5	11.9	0.833	9.2	10	10.5	10.2	0.405	12.4	12.4	13.2	12.8	0.525	13.6	11.8	13.1	11.8	0.397
	± 0.4	± 0.4	± 0.5	± 0.5		± 0.7	± 0.8	± 0.8	± 0.9		± 0.5	± 0.7	± 0.7	± 0.9		± 1.1	± 0.8	± 0.9	± 1.0	
**Total food purchases (%±SE)**																				
Convenience stores	1.5	1.5	1.6	1.6	0.261	1	1.4	1.4	1.3	0.606	1.4	1.3	1.4	1.5	0.291	2.1	2.2	2.4	2.2	0.744
	± 0.1	± 0.1	± 0.1	± 0.1		± 0.2	± 0.2	± 0.2	± 0.2		± 0.1	± 0.1	± 0.1	± 0.1		± 0.3	± 0.2	± 0.3	± 0.2	
Supermarkets	46.7	48	47.4	48.5	0.087	31.1	34.7 ± 1.2	36.2	38	<0.001	46.9	49	48	49	0.14	60.7	58.7	58	59.1	0.317
	± 0.5	± 0.6	± 0.6	± 0.7		± 1.1		± 1.3	± 1.4		± 0.7	± 0.8	± 0.8	± 0.9		± 1.0	± 1.0	± 1.1	± 1.4	
Wholesalers/price clubs	3.6	3.9	4.4	4.4	0.004	1.6	1.4 ± 0.3	1.7	1.6	0.822	2.5	3	4	3.8	<0.001	8.3	8.3	8.3	8.8	0.574
	± 0.2	± 0.2	± 0.2	± 0.3		± 0.4		± 0.3	± 0.3		± 0.2	± 0.3	± 0.3	± 0.4		± 0.6	± 0.5	± 0.6	± 0.8	
Traditional retailers	42.6	42.4	42	40.7	0.028	60.5	58.6 ± 1.2	56.4	54.7	0.001	43.7	42.8	42.4	40.9	0.018	23.1	25.9	25.4	24.9	0.341
	± 0.6	± 0.6	± 0.6	± 0.7		± 1.2		± 1.3	± 1.4		± 0.7	± 0.8	± 0.9	± 0.9		± 0.9	± 1.0	± 1.0	± 1.2	
Others	5.7	4.2	4.6	4.8	0.015	5.7	3.9 ± 0.4	4.2	4.5	0.120	5.6	4	4.3	4.8	0.070	5.8	5	5.9	5.1	0.470
	± 0.2	± 0.2	± 0.2	± 0.2		± 0.4		± 0.4	± 0.4		± 0.3	± 0.2	± 0.2	± 0.3		± 0.4	± 0.3	± 0.5	± 0.4	

Source: Authors’ own analyses and calculations based on data from Nielsen through its Mexico Consumer Panel Service (CPS) for the food and beverage categories for January 2012 to December 2015. The Nielsen Company, 2016. Nielsen is not responsible for and had no role in preparing the results reported herein. Multivariate linear regression models were used to predict unadjusted percentages of per capita daily purchases of total, taxed, and untaxed beverages and foods according to store-type, socioeconomic status (SES), and year of purchases. Percentages were weighted to be urban representative. Our statistical testing focused on the trends analysis.

## Supplementary Materials

The following are available online at http://www.mdpi.com/2072-6643/10/8/1044/s1. Table S1: Store-type categorization and examples of food stores included in each category, Table S2: Foods and beverages categories available in Nielsen CPS, Table S3: Unadjusted percentage mean of beverage purchases from 2012 to 2015 by store-type and socioeconomic status, Table S4: Unadjusted percentage mean of food purchases from 2012 to 2015 by store-type and socioeconomic status, Table S5: Unadjusted mean daily beverage purchases per capita from 2012 to 2015 by store-type and socioeconomic status, Table S6: Unadjusted mean daily food purchases per capita from 2012 to 2015 by store-type and socioeconomic status.
