# Supplementary material for: Mexican Households’ Purchases of Foods and Beverages Vary by Store-Type, Taxation Status, and SES
Source: Nutrients. 2018 Aug 8;10(8):1044. doi: 10.3390/nu10081044 (PMC6115932; doi:10.3390/nu10081044)
Supplement: Supplementary file 1 [file nutrients-10-01044-s001.pdf]

**Supplemental Table 1. Store-type categorization and examples of food stores included in each category**

| Store-type categories | Definition* | Examples of food stores in categories |
|---|---|---|
| Convenience stores | Chained retail outlets with a selling area of less than 400 $m^2$, and with a primary focus on selling food/beverages/tobacco and other groceries. | 7-eleven, Oxxo, Circulo K, Extra, etc. |
| Supermarkets | Usually chained, but also independent, retail outlets with a selling space from 400 $m^2$ to over 2500 $m^2$ and with a primary focus on selling food/beverages/tobacco and other groceries. Supermarkets also sell a range of non-groceries. | Wal-Mart, H-E-B, Comercial Mexicana, Chedraui, Soriana, Superama, City-Market, etc. |
| Wholesalers/price clubs | Retail outlets with an area larger than 4,500 m2 and sell groceries, perishables, clothing and general merchandise (furniture, gifts, personal care products, appliances, among others), in bulk or large packages. They offer additional services such as pharmacy, food court, among others. | Costco, Sam's Club |
| Traditional retailers | Typically, family owned independent retail outlets with a selling area of less than 400 $m^2$ and with a primary focus on selling food/beverages/tobacco and other groceries. Food usually accounts for at least 50% of total retail sales value. Includes non-chained bakeries, tortilla shops, peddlers and kiosks installed in permanent public markets. | Traditional shops, fixed markets or miscellany usually attended by the owner, so no chain names available. |
| Home water -delivery | Scheduled delivery, ranging from monthly to weekly or bi weekly, of one or more 5-gallon (20 liters) water jugs to households. This service is provided by multiple manufacturers, e.g. E-pura, Bonafont, Ciel, etc. | Delivery of 5-gallon water jugs |
| Others | Includes other types of stores that may sell specific packaged foods (e.g. candy, soda or water) but that combined account for <1% of purchases of foods and beverages. These stores main purpose is other than being a food outlet. E.g. movie theaters, pet-shops, etc. | Cinepolis, Liverpool, Farmacia del Ahorro, etc. |

*Store-type categories are based on the definitions provided by the Mexican National Association of Self-Service and Departmental Stores (Asociación Nacional de Tiendas de Autoservicio y Departamentales (ANTAD), in Spanish) and Euromonitor International, which consider the size of the property where stores are located, the merchandise they sell and the additional services they offer to consumers.

| **Supplemental Table 2. Foods and beverages categories available in Nielsen CPS** | | |
|---|---|---|
| **Foods** | | |
| **Taxation status*** | **Categorization** | **Products included** |
| Taxed | Salty snacks ≥275 Kcal/100g | Potato chips, corn chips, flour chips, fried pork skin, ready-to-eat popcorn, microwave popcorn, crackers, peanuts and seeds |
| | Cereal based sweets | Cookies, pre-packaged sweet bread and cakes, cereal bars |
| | Ready-to-eat cereals | Pre-prepared, ready-to-eat cereals |
| | Sweets and desserts | Ice-cream, ice-pops, sorbets, "dulce de leche" or "cajeta", fruit preserves, jellies, jam, milk modifiers with chocolate or other flavors |
| Untaxed | Salty snacks <275 kcal/100g | Crackers, flour chips, fried pork skin |
| | Cereals | Unsweetened breads and rolls, ready-to-eat cereals, pasta, corn, rice, oats |
| | Non-cereal based sweets | Ice cream, sorbets, popsicles, "dulce de leche" or "cajeta", fruit preserves, jellies, jam, gelatin, flan, pudding |
| | Dairy | Cheese, solid yogurt, cream, evaporated milk |
| | Processed fruits & vegetables | Canned vegetables, tomato puree, frozen fruit |
| | Sugar & sugar substitutes | Sugar, sugar substitutes or non-caloric sweeteners |
| **Beverages** | | |
| **Taxation status*** | **Categorization** | **Products included** |
| Taxed | Sugar sweetened sodas | Sodas |
| | Non-carbonated beverages with added sugar | Industrialized flavored waters with added sugar, tea, sports drinks |
| | Juices from concentrate | Sweetened juices |
| | Other sugary beverages | Soy and non-dairy base beverages, coffee |
| | Sugar sweetened milk and dairy | Sugar milk and dairy (yogurt), milk modifiers (e.g. coffee mate) |
| Untaxed | Artificially Sweetened Sodas | Sodas |
| | Sparkling, still, or plain water | Plain and sparkling water |
| | Non-carbonated beverages with non-caloric sweeteners | Industrialized flavored water with non-caloric sweeteners, tea |
| | 100% Juices | 100% fruit or vegetable juices |
| | Unsweetened milk and dairy | Plain milk and dairy (yogurt) |
| | Other unsweetened or artificially sweetened beverages | Soy and non-dairy base beverages, atole, coffee, milk modifiers, beer |
| *Mexican legislation taxes with 1 peso/liter all beverages with added sugar; and with an 8% tax all non-essential foods with ≥ 275kcal/100 g. A team of Mexican registered dieticians reviewed each product and assigned it into a tax and food/beverage category following the law definitions. Because products within one food/beverage group can have different characteristics under the Mexican legislation, food/beverages groups can appear under the tax and untaxed classifications. E.g. Salty snacks with ≥275kcal/100 g are classified as taxed, while salty snacks with <275kcal/100 g are classified as untaxed. | | |

**Supplemental Table 3.** Nielsen Company's Mexico Consumer Panel Services socioeconomic characteristics, sample sizes and proportion of households purchasing foods, beverages or both at every store-type from 2012 to 2015.

| Year | Overall | 2012 | 2013 | 2014 | 2015 |
|---|---|---|---|---|---|
| n | | 5,813 | 5,775 | 5,657 | 5,493 |
| **Socioeconomic strata ±SES, % ±SE** | | | | | |
| Low | 21.0 ±0.1 | 18.3 ±0.2 | 20.9 ±0.2 | 22.5 ±0.3 | 22.3 ±0.3 |
| Middle | 53.3 ±0.2 | 57.7 ±0.3 | 52.6 ±0.3 | 51.6 ±0.3 | 51.5 ±0.4 |
| High | 25.7 ±0.1 | 23.9 ±0.2 | 26.5 ±0.3 | 25.9 ±0.3 | 26.3 ±0.3 |
| **Household size (number of household members) mean ±SE** | 6.0 ±0.01 | 5.7 ±0.06 | 5.9 ±0.06 | 6.1 ±0.07 | 6.1 ±0.08 |
| **Households that purchased any foods and beverages at each retailer, % ±SE** | | | | | |
| Convenience stores | 75.4 ±0.5 | 73.9 ±0.9 | 74.9 ±0.9 | 75.0 ±0.9 | 77.5 ±1.0 |
| Supermarkets | 98.7 ±0.1 | 98.6 ±0.2 | 98.5 ±0.2 | 98.4 ±0.3 | 99.0 ±0.2 |
| Wholesaler/price clubs | 38.2 ±0.5 | 36.5 ±0.9 | 36.8 ±1.0 | 40.1 ±1.1 | 39.2 ±1.2 |
| Traditional retailers | 99.7 ±0.0 | 99.7 ±0.1 | 99.7 ±0.1 | 99.8 ±0.1 | 99.7 ±0.1 |
| Home water-delivery | 79.5 ±0.4 | 80.4 ±0.8 | 80.3 ±0.8 | 77.8 ±0.9 | 79.6 ±1.0 |
| Other | 89.5 ±0.3 | 92.1 ±0.5 | 89.1 ±0.7 | 88.1 ±0.7 | 88.9 ±0.7 |
| **Households that purchased beverages at each retailer, % ±SE** | | | | | |
| Convenience stores | 70.1 ±0.5 | 68.0 ±0.9 | 69.9 ±0.9 | 69.7 ±1.0 | 72.9 ±1.1 |
| Supermarkets | 96.5 ±0.2 | 96.7 ±0.3 | 96.7 ±0.4 | 96.3 ±0.4 | 96.4 ±0.4 |
| Wholesaler/price clubs | 27.9 ±0.5 | 26.1 ±0.8 | 27.2 ±0.9 | 29.6 ±1.0 | 28.7 ±1.2 |
| Traditional retailers | 99.5 ±0.1 | 99.3 ±0.1 | 99.5 ±0.1 | 99.6 ±0.1 | 99.5 ±0.1 |
| Home water-delivery | 75.8 ±0.5 | 76.8 ±0.8 | 76.0 ±0.9 | 74.5 ±0.9 | 75.8 ±1.1 |
| Other | 80.3 ±0.4 | 84.0 ±0.7 | 80.0 ±0.8 | 78.1 ±0.9 | 79.2 ±1.0 |
| **Households that purchased food at each retailer, % ±SE** | | | | | |
| Convenience stores | 51.5 ±0.5 | 49.8 ±1.0 | 50.6 ±1.0 | 51.3 ±1.1 | 54.0 ±1.2 |
| Supermarkets | 98.1 ±0.1 | 98.0 ±0.3 | 98.1 ±0.3 | 97.5 ±0.4 | 98.8 ±0.2 |
| Wholesaler/price clubs | 34.0 ±0.5 | 32.2 ±0.9 | 32.8 ±1.0 | 35.6 ±1.1 | 35.5 ±1.2 |
| Traditional retailers | 99.2 ±0.1 | 99.1 ±0.2 | 99.3 ±0.1 | 99.1 ±0.2 | 99.1 ±0.2 |
| Other | 78.2 ±0.4 | 83.0 ±0.8 | 76.4 ±0.9 | 75.8 ±0.9 | 77.7 ±1.0 |

Source: Authors' own analyses and calculations based on data from Nielsen through its Mexico Consumer Panel Service (CPS) for the food and beverage categories for January 2012 – December 2015. The Nielsen Company, 2016. Nielsen is not responsible for and had no role in preparing the results reported herein. SES classification is based on the socioeconomic index provided by Nielsen.

**Supplemental Table 3. Unadjusted percentage mean of taxed and untaxed beverage purchases from 2012 to 2015 by store-type and socioeconomic status**

| | Total population | | | | P for trend | Low SES | | | | P for trend | Medium SES | | | | P for trend | High SES | | | | P for trend |
|---|---|---|---|---|---|---|---|---|---|---|---|---|---|---|---|---|---|---|---|---|
| | 2012 | 2013 | 2014 | 2015 | | 2012 | 2013 | 2014 | 2015 | | 2012 | 2013 | 2014 | 2015 | | 2012 | 2013 | 2014 | 2015 | |
| | n=5813 | n=5775 | n=5657 | n=5493 | | n=959 | n=1094 | n=1087 | n=1170 | | n=3133 | n=2872 | n=2815 | n=2690 | | n=1721 | n=1809 | n=1755 | n=1633 | |
| **Taxed beverage purchases (%±SE)** | | | | | | | | | | | | | | | | | | | | |
| Convenience stores | 2.0 ± 0.2 | 2.3 ± 0.2 | 2.6 ± 0.2 | 2.9 ± 0.2 | <0.001 | 0.7 ± 0.1 | 1.0 ± 0.2 | 1.1 ± 0.2 | 1.1 ± 0.2 | 0.160 | 1.6 ± 0.2 | 2.0 ± 0.2 | 2.2 ± 0.2 | 2.4 ± 0.2 | 0.011 | 4.3 ± 0.5 | 4.4 ± 0.5 | 5.1 ± 0.6 | 5.9 ± 0.6 | 0.019 |
| Supermarkets | 21.6 ± 0.5 | 21.9 ± 0.5 | 20.3 ± 0.5 | 19.4 ± 0.6 | 0.001 | 11.3 ± 0.7 | 12.7 ± 0.8 | 12.1 ± 0.8 | 11.4 ± 0.9 | 0.893 | 19.7 ± 0.6 | 20.5 ± 0.7 | 19.1 ± 0.7 | 17.7 ± 0.7 | 0.015 | 36.5 ± 1.2 | 33.8 ± 1.2 | 32.1 ± 1.2 | 32.1 ± 1.4 | 0.012 |
| Wholesalers/price clubs | 2.1 ± 0.2 | 1.9 ± 0.1 | 1.9 ± 0.2 | 1.6 ± 0.2 | 0.019 | 0.5 ± 0.1 | 0.4 ± 0.1 | 0.5 ± 0.1 | 0.4 ± 0.1 | 0.673 | 1.2 ± 0.1 | 1.2 ± 0.2 | 1.6 ± 0.3 | 1.2 ± 0.2 | 0.645 | 6.0 ± 0.6 | 4.9 ± 0.4 | 4.0 ± 0.4 | 3.6 ± 0.5 | 0.001 |
| Traditional retailers | 70.8 ± 0.6 | 70.8 ± 0.6 | 71.9 ± 0.7 | 72.5 ± 0.7 | 0.031 | 85.6 ± 0.8 | 83.6 ± 0.9 | 84.4 ± 1 | 85.2 ± 1 | 0.978 | 74.6 ± 0.7 | 73.8 ± 0.8 | 74.1 ± 0.9 | 75.0 ± 1.0 | 0.689 | 46.9 ± 1.4 | 51.8 ± 1.4 | 53.6 ± 1.5 | 53.3 ± 1.7 | 0.003 |
| Home water-delivery | 1.0 ± 0.1 | 1.1 ± 0.1 | 1.3 ± 0.1 | 1.1 ± 0.1 | 0.39 | 0.3 ± 0.1 | 0.5 ± 0.1 | 0.5 ± 0.1 | 0.4 ± 0.2 | 0.754 | 0.8 ± 0.1 | 1.0 ± 0.1 | 1.2 ± 0.2 | 1.1 ± 0.2 | 0.157 | 2.2 ± 0.3 | 2.1 ± 0.3 | 2.4 ± 0.4 | 2.0 ± 0.4 | 0.885 |
| Others | 2.4 ± 0.2 | 1.9 ± 0.2 | 2.1 ± 0.2 | 2.5 ± 0.3 | 0.796 | 1.6 ± 0.2 | 1.8 ± 0.6 | 1.5 ± 0.3 | 1.5 ± 0.2 | 0.507 | 2.1 ± 0.3 | 1.5 ± 0.2 | 2.0 ± 0.2 | 2.7 ± 0.5 | 0.227 | 4.1 ± 0.5 | 2.9 ± 0.3 | 2.9 ± 0.3 | 3.1 ± 0.5 | 0.184 |
| **Untaxed beverage purchases (%±SE)** | | | | | | | | | | | | | | | | | | | | |
| Convenience stores | 5.2 ± 0.2 | 5.5 ± 0.3 | 5.6 ± 0.3 | 5.7 ± 0.4 | 0.276 | 6.8 ± 0.7 | 6.6 ± 0.6 | 6.2 ± 0.6 | 5.7 ± 0.8 | 0.251 | 4.9 ± 0.3 | 4.8 ± 0.3 | 5.2 ± 0.4 | 5.4 ± 0.5 | 0.381 | 4.6 ± 0.4 | 5.9 ± 0.6 | 6.0 ± 0.6 | 6.5 ± 0.8 | 0.068 |
| Supermarkets | 11.0 ± 0.4 | 11.4 ± 0.4 | 11.7 ± 0.4 | 11.6 ± 0.5 | 0.268 | 7.2 ± 0.7 | 8.3 ± 0.7 | 8.6 ± 0.7 | 9.0 ± 0.9 | 0.115 | 10.6 ± 0.5 | 11.2 ± 0.5 | 11.7 ± 0.6 | 10.9 ± 0.7 | 0.551 | 15.3 ± 0.8 | 14.9 ± 0.8 | 14.9 ± 0.8 | 15.8 ± 1.1 | 0.729 |
| Wholesalers/price clubs | 0.7 ± 0.1 | 0.8 ± 0.1 | 0.9 ± 0.1 | 1.1 ± 0.2 | 0.012 | 0.2 ± 0.1 | 0.3 ± 0.1 | 0.3 ± 0.1 | 0.3 ± 0.1 | 0.752 | 0.4 ± 0.1 | 0.5 ± 0.1 | 0.7 ± 0.1 | 0.7 ± 0.2 | 0.014 | 2.0 ± 0.2 | 2.2 ± 0.3 | 2.1 ± 0.2 | 2.8 ± 0.6 | 0.176 |
| Traditional retailers | 26.1 ± 0.6 | 26.6 ± 0.6 | 26.2 ± 0.6 | 25.1 ± 0.7 | 0.215 | 32.3 ± 1.3 | 31.2 ± 1.3 | 30.5 ± 1.4 | 29.1 ± 1.5 | 0.105 | 26.1 ± 0.7 | 27.8 ± 0.8 | 27.3 ± 0.9 | 26.4 ± 0.9 | 0.885 | 20.3 ± 1.1 | 19.4 ± 1.1 | 19.0 ± 1.0 | 17.8 ± 1.2 | 0.116 |
| Home water-delivery | 42.4 ± 0.7 | 41.7 ± 0.7 | 40.9 ± 0.8 | 42.8 ± 0.9 | 0.866 | 42.1 ± 1.5 | 41.3 ± 1.6 | 41.6 ± 1.6 | 43.8 ± 1.8 | 0.433 | 42.5 ± 0.9 | 40.8 ± 1.0 | 39.5 ± 1.1 | 41.7 ± 1.3 | 0.439 | 42.2 ± 1.5 | 44.0 ± 1.5 | 43.0 ± 1.6 | 44.2 ± 1.9 | 0.505 |
| Others | 14.6 ± 0.5 | 14.1 ± 0.5 | 14.6 ± 0.6 | 13.7 ± 0.6 | 0.367 | 11.4 ± 0.8 | 12.4 ± 1.0 | 12.6 ± 1.0 | 12.1 ± 1.0 | 0.630 | 15.4 ± 0.7 | 15 ± 0.8 | 15.5 ± 0.9 | 14.8 ± 1.0 | 0.733 | 15.6 ± 1.2 | 13.6 ± 1.0 | 14.9 ± 1.1 | 12.9 ± 1.1 | 0.192 |

Source: Authors' own analyses and calculations based on data from Nielsen through its Mexico Consumer Panel Service (CPS) for the food and beverage categories for January 2012 – December 2015. The Nielsen Company, 2016. Nielsen is not responsible for and had no role in preparing the results reported herein. Multivariate linear regression models were used to predict unadjusted percentages of per capita daily purchases of taxed and untaxed beverages according to store-type, SES and year of purchases. Percentages were weighted to be urban representative. Our statistical testing focused on the trends analysis.

**Supplemental Table 4. Unadjusted percentage mean of taxed and untaxed food purchases from 2012 to 2015 by store-type and socioeconomic status**

| | Total population | | | | | Low SES | | | | | Medium SES | | | | | High SES | | | | |
|---|---|---|---|---|---|---|---|---|---|---|---|---|---|---|---|---|---|---|---|---|
| | 2012 | 2013 | 2014 | 2015 | P for trend | 2012 | 2013 | 2014 | 2015 | P for trend | 2012 | 2013 | 2014 | 2015 | P for trend | 2012 | 2013 | 2014 | 2015 | P for trend |
| | n=5813 | n=5775 | n=5657 | n=5493 | | n=959 | n=1094 | n=1087 | n=1170 | | n=3133 | n=2872 | n=2815 | n=2690 | | n=1721 | n=1809 | n=1755 | n=1633 | |
| **Taxed food purchases (%±SE)** | | | | | | | | | | | | | | | | | | | | |
| Convenience stores | 2.3 ± 0.1 | 2.5 ± 0.1 | 2.7 ± 0.2 | 2.5 ± 0.1 | 0.170 | 1.5 ± 0.2 | 2.0 ± 0.3 | 2.2 ± 0.4 | 1.8 ± 0.2 | 0.365 | 2.2 ± 0.1 | 2.3 ± 0.1 | 2.5 ± 0.2 | 2.5 ± 0.2 | 0.105 | 3.5 ± 0.4 | 3.7 ± 0.4 | 3.9 ± 0.5 | 3.5 ± 0.4 | 0.984 |
| Supermarkets | 46 ± 0.6 | 46 ± 0.6 | 46.1 ± 0.7 | 48.4 ± 0.8 | 0.014 | 27.1 ± 1.1 | 30.4 ± 1.2 | 32.1 ± 1.3 | 37.4 ± 1.5 | <0.001 | 46.6 ± 0.8 | 46.8 ± 0.8 | 47.0 ± 0.9 | 48.7 ± 1.1 | 0.119 | 61.8 ± 1.1 | 59.4 ± 1.1 | 59.2 ± 1.2 | 59.9 ± 1.5 | 0.333 |
| Wholesalers/price clubs | 4.4 ± 0.2 | 4.8 ± 0.3 | 5.4 ± 0.3 | 5.8 ± 0.4 | 0.001 | 1.5 ± 0.4 | 1.4 ± 0.3 | 1.7 ± 0.3 | 1.6 ± 0.3 | 0.664 | 3.0 ± 0.2 | 3.5 ± 0.3 | 4.5 ± 0.4 | 4.9 ± 0.5 | <0.001 | 10.9 ± 0.7 | 11.0 ± 0.7 | 11.2 ± 0.7 | 12.1 ± 1 | 0.288 |
| Traditional retailers | 43.1 ± 0.6 | 43.9 ± 0.7 | 42.4 ± 0.7 | 39.7 ± 0.8 | <0.001 | 65.6 ± 1.2 | 63.7 ± 1.3 | 61.0 ± 1.4 | 55.7 ± 1.5 | <0.001 | 44.1 ± 0.8 | 44.7 ± 0.9 | 42.6 ± 0.9 | 40.1 ± 1.1 | 0.001 | 19.5 ± 1.0 | 22.8 ± 1.1 | 21.9 ± 1.1 | 21.3 ± 1.3 | 0.440 |
| Others | 4.2 ± 0.2 | 2.7 ± 0.1 | 3.2 ± 0.2 | 3.3 ± 0.3 | 0.068 | 4.3 ± 0.4 | 2.5 ± 0.3 | 2.7 ± 0.3 | 3.1 ± 0.5 | 0.152 | 4.2 ± 0.3 | 2.7 ± 0.2 | 3.2 ± 0.3 | 3.5 ± 0.6 | 0.414 | 4.3 ± 0.3 | 3.1 ± 0.3 | 3.8 ± 0.4 | 3.1 ± 0.3 | 0.053 |
| **Untaxed food purchases (%±SE)** | | | | | | | | | | | | | | | | | | | | |
| Convenience stores | 1.2 ± 0.1 | 1.3 ± 0.1 | 1.3 ± 0.1 | 1.3 ± 0.1 | 0.279 | 0.9 ± 0.2 | 1.2 ± 0.3 | 1. 2 ± 0.2 | 1.2 ± 0.3 | 0.564 | 1.1 ± 0.1 | 1.0 ± 0.1 | 1.1 ± 0.1 | 1.3 ± 0.1 | 0.390 | 1.7 ± 0.3 | 1.8 ± 0.2 | 2.0 ± 0.3 | 1.8 ± 0.2 | 0.736 |
| Supermarkets | 47.4 ± 0.6 | 48.9 ± 0.6 | 48.1 ± 0.6 | 48.9 ± 0.7 | 0.193 | 32.6 ± 1.2 | 36.3 ± 1.2 | 37.5 ± 1.3 | 38.6 ± 1.4 | 0.002 | 47.5 ± 0.7 | 50.2 ± 0.8 | 48.8 ± 0.8 | 49.5 ± 1 | 0.204 | 60.7 ± 1.1 | 58.7 ± 1 | 57.9 ± 1.1 | 59.0 ± 1.4 | 0.307 |
| Wholesalers/price clubs | 3.3 ± 0.2 | 3.6 ± 0.2 | 4.1 ± 0.2 | 4.0 ± 0.3 | 0.016 | 1.7 ± 0.4 | 1.4 ± 0.3 | 1.7 ± 0.3 | 1.6 ± 0.3 | 0.938 | 2.3 ± 0.2 | 2.8 ± 0.3 | 3.8 ± 0.3 | 3.4 ± 0.4 | 0.001 | 7.4 ± 0.5 | 7.4 ± 0.5 | 7.5 ± 0.6 | 7.8 ± 0.8 | 0.687 |
| Traditional retailers | 42.0 ± 0.6 | 41.6 ± 0.6 | 41.4 ± 0.6 | 40.6 ± 0.7 | 0.136 | 58.7 ± 1.2 | 56.8 ± 1.3 | 54.9 ± 1.3 | 53.9 ± 1.5 | 0.008 | 42.9 ± 0.7 | 41.8 ± 0.8 | 41.7 ± 0.9 | 40.7 ± 1 | 0.070 | 23.9 ± 0.9 | 26.6 ± 1.0 | 26.2 ± 1.1 | 25.9 ± 1.2 | 0.280 |
| Others | 6.2 ± 0.2 | 4.6 ± 0.2 | 5.0 ± 0.2 | 5.2 ± 0.2 | 0.011 | 6.1 ± 0.4 | 4.3 ± 0.4 | 4.6 ± 0.5 | 4.8 ± 0.5 | 0.126 | 6.1 ± 0.3 | 4.3 ± 0.2 | 4.6 ± 0.3 | 5.2 ± 0.3 | 0.040 | 6.3 ± 0.4 | 5.5 ± 0.4 | 6.4 ± 0.6 | 5.6 ± 0.5 | 0.528 |

Source: Authors' own analyses and calculations based on data from Nielsen through its Mexico Consumer Panel Service (CPS) for the food and beverage categories for January 2012 – December 2015. The Nielsen Company, 2016. Nielsen is not responsible for and had no role in preparing the results reported herein. Multivariate linear regression models were used to predict unadjusted percentages of per capita daily purchases of taxed and untaxed foods according to store-type, SES and year of purchases. Percentages were weighted to be urban representative. Our statistical testing focused on the trends analysis.

**Supplemental Table 5. Unadjusted mean daily beverage purchases per capita from 2012 to 2015 by store-type and socioeconomic status**

| | Total population | | | | P for trend | Low SES | | | | P for trend | Medium SES | | | | P for trend | High SES | | | | P for trend |
|---|---|---|---|---|---|---|---|---|---|---|---|---|---|---|---|---|---|---|---|---|
| | 2012 n=5813 | 2013 n=5775 | 2014 n=5657 | 2015 n=5493 | | 2012 n=959 | 2013 n=1094 | 2014 n=1087 | 2015 n=1170 | | 2012 n=3133 | 2013 n=2872 | 2014 n=2815 | 2015 n=2690 | | 2012 n=1721 | 2013 n=1809 | 2014 n=1755 | 2015 n=1633 | |
| **Total beverage purchases (mL/capita/day) (mean ± SE)** | | | | | | | | | | | | | | | | | | | | |
| Convenience stores | 34.9 ± 1.6 | 40.1 ± 1.9 | 41.2 ± 2.2 | 40.6 ± 2.7 | 0.064 | 36.9 ± 3 | 39.9 ± 3.1 | 35.2 ± 2.8 | 31.7 ± 3.2 | 0.237 | 33.1 ± 2.1 | 35.4 ± 2.1 | 38.3 ± 3.1 | 38.7 ± 3.3 | 0.103 | 37.8 ± 3.6 | 50.8 ± 5.8 | 54.1 ± 5.9 | 54.5 ± 7.9 | 0.061 |
| Supermarkets | 86.4 ± 2 | 96.6 ± 2.4 | 90.4 ± 2.6 | 79.3 ± 2.7 | 0.01 | 45.8 ± 3.3 | 56.0 ± 3.7 | 50.7 ± 3.6 | 47.7 ± 4.1 | 0.709 | 81.0 ± 2.5 | 92.5 ± 3.1 | 88.2 ± 3.3 | 75.0 ± 3.5 | 0.123 | 138.3 ± 5.1 | 145.3 ± 6 | 138.0 ± 6.8 | 123.5 ± 6.9 | 0.052 |
| Wholesalers/price clubs | 7.7 ± 0.6 | 9.0 ± 0.8 | 8.3 ± 0.8 | 7.5 ± 0.7 | 0.612 | 1.6 ± 0.4 | 2.5 ± 0.7 | 2.3 ± 0.5 | 2.0 ± 0.5 | 0.454 | 4.1 ± 0.5 | 5.6 ± 1.1 | 6.9 ± 1.3 | 5.2 ± 0.8 | 0.123 | 23.2 ± 2.5 | 22.9 ± 2.2 | 18.1 ± 1.7 | 18.3 ± 2.5 | 0.081 |
| Traditional retailers | 350.8 ± 7.8 | 356.3 ± 8.3 | 338.6 ± 7.8 | 327.7 ± 9.1 | 0.021 | 373.8 ± 15.5 | 374.0 ± 16.3 | 355.0 ± 17.4 | 345.0 ± 17.5 | 0.217 | 367.4 ± 10.4 | 381.2 ± 12.2 | 359.3 ± 10.5 | 355.7 ± 13.8 | 0.306 | 285.1 ± 17.7 | 284.2 ± 14.6 | 275.3 ± 15.3 | 246.7 ± 14.6 | 0.077 |
| Home water-delivery | 466.9 ± 11.7 | 476.1 ± 12.1 | 486.0 ± 14.3 | 485 ± 15.1 | 0.293 | 440.8 ± 24.7 | 438.7 ± 23.9 | 461.4 ± 28.5 | 469.4 ± 27.3 | 0.437 | 478.2 ± 16.6 | 468.8 ± 17.4 | 476.6 ± 19.6 | 491.5 ± 22.8 | 0.597 | 461.0 ± 20.6 | 528.7 ± 22.8 | 533.0 ± 30.4 | 487.6 ± 28.6 | 0.517 |
| Others | 152 ± 7.1 | 158.7 ± 8.5 | 169.9 ± 9.2 | 146.3 ± 8.4 | 0.836 | 120.5 ± 15.9 | 133.0 ± 17.1 | 127.6 ± 12.7 | 110.0 ± 13.3 | 0.612 | 156.5 ± 8.7 | 168.0 ± 12.7 | 188.6 ± 15.1 | 161.3 ± 12.7 | 0.476 | 169.3 ± 18 | 163.4 ± 13.9 | 174.5 ± 15.8 | 152.8 ± 16.9 | 0.609 |
| **Taxed beverage purchases (mL/capita/day) (mean ± SE)** | | | | | | | | | | | | | | | | | | | | |
| Convenience stores | 4.4 ± 0.5 | 4.8 ± 0.6 | 4.5 ± 0.4 | 4.5 ± 0.4 | 0.925 | 1.8 ± 0.4 | 2.1 ± 0.4 | 2.2 ± 0.8 | 2.5 ± 0.9 | 0.486 | 3.9 ± 0.7 | 4.5 ± 0.9 | 4.0 ± 0.4 | 4.4 ± 0.6 | 0.709 | 8.1 ± 1.2 | 8.0 ± 1.2 | 8.2 ± 1.3 | 7.1 ± 0.9 | 0.538 |
| Supermarkets | 36.6 ± 1 | 36.7 ± 1.1 | 28.9 ± 1 | 24.2 ± 1 | <0.001 | 20.6 ± 1.8 | 22.3 ± 1.9 | 16.9 ± 1.7 | 16.1 ± 2 | 0.022 | 34.6 ± 1.3 | 36.0 ± 1.6 | 29.2 ± 1.5 | 24.0 ± 1.3 | <0.001 | 56.8 ± 2.4 | 52.2 ± 2.6 | 41.3 ± 2.2 | 33.7 ± 2.1 | <0.001 |
| Wholesalers/price clubs | 3.9 ± 0.4 | 3.6 ± 0.4 | 2.6 ± 0.3 | 1.7 ± 0.2 | <0.001 | 0.9 ± 0.3 | 0.8 ± 0.3 | 1.0 ± 0.3 | 0.6 ± 0.2 | 0.473 | 2.1 ± 0.3 | 2.5 ± 0.6 | 2.4 ± 0.4 | 1.5 ± 0.2 | 0.106 | 11.4 ± 1.6 | 8.5 ± 1.2 | 4.7 ± 0.5 | 3.5 ± 0.5 | <0.001 |
| Traditional retailers | 173.7 ± 3.4 | 166.9 ± 3.4 | 151.3 ± 3.4 | 141.7 ± 3.8 | <0.001 | 206.2 ± 8.1 | 188.2 ± 7.1 | 168.9 ± 7.3 | 160.8 ± 7.9 | <0.001 | 186.9 ± 4.5 | 177.3 ± 4.7 | 159.0 ± 4.5 | 151.4 ± 5.3 | <0.001 | 108.5 ± 5.4 | 123.3 ± 7.0 | 115.2 ± 7.3 | 99.1 ± 7.2 | 0.175 |
| Home water-delivery | 1.9 ± 0.3 | 1.9 ± 0.2 | 2.1 ± 0.3 | 2 ± 0.4 | 0.619 | 0.7 ± 0.1 | 0.8 ± 0.1 | 0.5 ± 0.1 | 0.9 ± 0.3 | 0.713 | 1.8 ± 0.4 | 1.8 ± 0.3 | 2.2 ± 0.4 | 2.1 ± 0.7 | 0.635 | 3.1 ± 0.4 | 3 ± 0.4 | 3.5 ± 0.7 | 3.3 ± 1.1 | 0.771 |
| Others | 3.8 ± 0.2 | 3.1 ± 0.3 | 3.0 ± 0.3 | 3.5 ± 0.7 | 0.683 | 2.9 ± 0.5 | 2.6 ± 0.7 | 2.4 ± 0.5 | 1.9 ± 0.3 | 0.071 | 3.5 ± 0.3 | 2.8 ± 0.4 | 3.1 ± 0.4 | 4.7 ± 1.3 | 0.37 | 5.3 ± 0.6 | 4.4 ± 0.6 | 3.5 ± 0.5 | 2.7 ± 0.3 | <0.001 |
| **Untaxed beverage purchases (mL/capita/day) (mean ± SE)** | | | | | | | | | | | | | | | | | | | | |
| Convenience stores | 30.5 ± 1.4 | 35.3 ± 1.7 | 36.7 ± 2.1 | 36.1 ± 2.6 | 0.054 | 35.1 ± 3 | 37.9 ± 3.1 | 33.0 ± 2.7 | 29.2 ± 3.1 | 0.078 | 29.2 ± 1.9 | 30.8 ± 1.8 | 34.3 ± 3 | 34.3 ± 3.2 | 0.106 | 29.6 ± 2.9 | 42.8 ± 5.3 | 45.8 ± 5.4 | 47.4 ± 7.7 | 0.037 |
| Supermarkets | 49.8 ± 1.4 | 59.9 ± 1.7 | 61.5 ± 2 | 55.1 ± 2.2 | 0.042 | 25.2 ± 2.2 | 33.7 ± 2.4 | 33.8 ± 2.6 | 31.6 ± 2.9 | 0.135 | 46.4 ± 1.8 | 56.5 ± 2.2 | 59.1 ± 2.4 | 51.0 ± 2.8 | 0.103 | 81.5 ± 3.8 | 93.1 ± 4.5 | 96.7 ± 5.5 | 89.9 ± 5.7 | 0.218 |
| Wholesalers/price clubs | 3.9 ± 0.4 | 5.4 ± 0.5 | 5.8 ± 0.7 | 5.7 ± 0.7 | 0.017 | 0.7 ± 0.2 | 1.7 ± 0.6 | 1.2 ± 0.4 | 1.5 ± 0.4 | 0.31 | 2.0 ± 0.3 | 3.1 ± 0.6 | 4.5 ± 1.1 | 3.8 ± 0.7 | 0.008 | 11.9 ± 1.4 | 14.3 ± 1.5 | 13.4 ± 1.4 | 14.8 ± 2.4 | 0.363 |
| Traditional retailers | 177.1 ± 6.4 | 189.4 ± 6.9 | 187.3 ± 6.3 | 186.1 ± 7.7 | 0.439 | 167.6 ± 11.4 | 185.8 ± 13.1 | 186.1 ± 14.1 | 184.1 ± 15.2 | 0.455 | 180.5 ± 8.6 | 203.9 ± 10.6 | 200.3 ± 8.6 | 204.3 ± 11.8 | 0.129 | 176.6 ± 15.3 | 160.9 ± 11 | 160.1 ± 12 | 147.6 ± 11.7 | 0.144 |
| Home water-delivery | 465 ± 11.7 | 474.2 ± 12.1 | 483.8 ± 14.3 | 483.0 ± 15.1 | 0.297 | 440.1 ± 24.7 | 437.9 ± 23.9 | 460.9 ± 28.4 | 468.5 ± 27.2 | 0.344 | 476.4 ± 16.5 | 467.0 ± 17.4 | 474.4 ± 19.6 | 489.4 ± 22.7 | 0.606 | 457.9 ± 20.5 | 525.8 ± 22.7 | 529.5 ± 30.1 | 484.4 ± 28.4 | 0.521 |

| | | | | | | | | | | | | | | | | | | | | |
|---|---|---|---|---|---|---|---|---|---|---|---|---|---|---|---|---|---|---|---|---|
| Others | 148.2 ± 7.1 | 155.6 ± 8.5 | 166.9 ± 9.2 | 142.8 ± 8.3 | 0.855 | 117.6 ± 15.9 | 130.4 ± 17.1 | 125.2 ± 12.7 | 108.2 ± 13.3 | 0.548 | 153.0 ± 8.6 | 165.2 ± 12.7 | 185.4 ± 15.1 | 156.6 ± 12.4 | 0.519 | 164 ± 17.9 | 159.0 ± 13.8 | 171 ± 15.6 | 150.2 ± 16.9 | 0.691 |

Source: Authors' own analyses and calculations based on data from Nielsen through its Mexico Consumer Panel Service (CPS) for the food and beverage categories for January 2012 – December 2015. The Nielsen Company, 2016. Nielsen is not responsible for and had no role in preparing the results reported herein. Multivariate linear regression models were used to predict unadjusted means of per capita daily purchases of taxed and untaxed beverages according to store-type, SES and year of purchases. Means were weighted to be urban representative. Our statistical testing focused on the trends analysis.

**Supplemental Table 6. Unadjusted mean daily food purchases per capita from 2012 to 2015 by store-type and socioeconomic status**

| | **Total population** | | | | | **Low SES** | | | | | **Medium SES** | | | | | **High SES** | | | | |
|---|---|---|---|---|---|---|---|---|---|---|---|---|---|---|---|---|---|---|---|---|
| | 2012 n=5813 | 2013 n=5775 | 2014 n=5657 | 2015 n=5493 | P for trend | 2012 n=959 | 2013 n=1094 | 2014 n=1087 | 2015 n=1170 | P for trend | 2012 n=3133 | 2013 n=2872 | 2014 n=2815 | 2015 n=2690 | P for trend | 2012 n=1721 | 2013 n=1809 | 2014 n=1755 | 2015 n=1633 | P for trend |
| **Total food purchases (g/capita/day) (mean ± SE)** | | | | | | | | | | | | | | | | | | | | |
| Convenience stores | 0.9 ± 0.1 | 1.0 ± 0.1 | 1.0 ± 0.1 | 0.9 ± 0.1 | 0.682 | 0.5 ± 0.1 | 0.7 ± 0.1 | 0.6 ± 0.1 | 0.6 ± 0.1 | 0.982 | 0.8 ± 0.1 | 0.9 ± 0.1 | 0.9 ± 0.1 | 0.9 ± 0.1 | 0.627 | 1.5 ± 0.2 | 1.5 ± 0.2 | 1.6 ± 0.2 | 1.3 ± 0.1 | 0.262 |
| Supermarkets | 32.5 ± 0.6 | 33.6 ± 0.7 | 32.5 ± 0.7 | 29.3 ± 0.7 | <0.001 | 17.3 ± 0.9 | 19.5 ± 1 | 19.8 ± 1.1 | 18.8 ± 1.3 | 0.429 | 31.2 ± 0.8 | 33.6 ± 1 | 32.6 ± 1 | 29.2 ± 1 | 0.087 | 50.2 ± 1.5 | 47.2 ± 1.4 | 45.9 ± 1.5 | 41.0 ± 1.6 | <0.001 |
| Wholesalers/price clubs | 2.8 ± 0.2 | 3.1 ± 0.2 | 3.5 ± 0.2 | 3.0 ± 0.2 | 0.406 | 0.8 ± 0.2 | 0.9 ± 0.2 | 0.9 ± 0.2 | 0.8 ± 0.1 | 0.88 | 1.8 ± 0.2 | 2.2 ± 0.3 | 3.0 ± 0.3 | 2.5 ± 0.3 | 0.006 | 7.5 ± 0.6 | 7.2 ± 0.5 | 7.4 ± 0.6 | 6.4 ± 0.8 | 0.311 |
| Traditional retailers | 23.4 ± 0.4 | 23 ± 0.4 | 21.7 ± 0.4 | 19.0 ± 0.4 | <0.001 | 29.6 ± 0.9 | 27.8 ± 0.9 | 25.7 ± 0.9 | 22.5 ± 0.9 | <0.001 | 24.2 ± 0.5 | 23.7 ± 0.6 | 22.4 ± 0.6 | 19.6 ± 0.6 | <0.001 | 15.6 ± 0.6 | 16.7 ± 0.7 | 15.8 ± 0.7 | 13.6 ± 0.7 | 0.019 |
| Others | 3.3 ± 0.1 | 2.5 ± 0.1 | 2.7 ± 0.2 | 2.3 ± 0.1 | <0.001 | 2.8 ± 0.3 | 1.9 ± 0.2 | 1.9 ± 0.2 | 1.7 ± 0.2 | 0.001 | 3.2 ± 0.2 | 2.2 ± 0.1 | 2.4 ± 0.2 | 2.3 ± 0.1 | <0.001 | 4.3 ± 0.3 | 3.6 ± 0.3 | 4.2 ± 0.5 | 3.0 ± 0.3 | 0.02 |
| **Taxed food purchases (g/capita/day) (mean ± SE)** | | | | | | | | | | | | | | | | | | | | |
| Convenience stores | 0.3 ± 0 | 0.4 ± 0 | 0.4 ± 0 | 0.3 ± 0 | 0.237 | 0.2 ± 0 | 0.3 ± 0 | 0.2 ± 0 | 0.2 ± 0 | 0.407 | 0.3 ± 0 | 0.4 ± 0 | 0.4 ± 0 | 0.3 ± 0 | 0.652 | 0.6 ± 0.1 | 0.6 ± 0.1 | 0.6 ± 0.1 | 0.5 ± 0.1 | 0.096 |
| Supermarkets | 7.3 ± 0.2 | 7.2 ± 0.2 | 6.8 ± 0.2 | 6.0 ± 0.2 | <0.001 | 3.6 ± 0.2 | 4.1 ± 0.2 | 3.9 ± 0.2 | 3.9 ± 0.3 | 0.590 | 7.1 ± 0.2 | 7.2 ± 0.2 | 6.8 ± 0.2 | 5.9 ± 0.2 | <0.001 | 11.3 ± 0.4 | 10.5 ± 0.4 | 9.7 ± 0.4 | 8.5 ± 0.4 | <0.001 |
| Wholesalers/price clubs | 0.8 ± 0 | 0.9 ± 0.1 | 1.0 ± 0.1 | 0.8 ± 0.1 | 0.491 | 0.2 ± 0 | 0.2 ± 0.1 | 0.2 ± 0 | 0.2 ± 0 | 0.717 | 0.5 ± 0 | 0.7 ± 0.1 | 0.9 ± 0.1 | 0.7 ± 0.1 | 0.028 | 2.2 ± 0.2 | 2.1 ± 0.1 | 2.2 ± 0.2 | 1.9 ± 0.2 | 0.335 |
| Traditional retailers | 6.1 ± 0.1 | 6.1 ± 0.1 | 5.3 ± 0.1 | 4.3 ± 0.1 | <0.001 | 8.6 ± 0.3 | 8.0 ± 0.3 | 6.8 ± 0.3 | 5.3 ± 0.3 | <0.001 | 6.4 ± 0.2 | 6.3 ± 0.2 | 5.5 ± 0.2 | 4.6 ± 0.2 | <0.001 | 3.1 ± 0.2 | 3.7 ± 0.3 | 3.3 ± 0.2 | 2.6 ± 0.2 | 0.033 |
| Others | 0.5 ± 0 | 0.4 ± 0 | 0.4 ± 0 | 0.3 ± 0 | <0.001 | 0.5 ± 0.1 | 0.3 ± 0.1 | 0.2 ± 0 | 0.2 ± 0 | <0.001 | 0.5 ± 0 | 0.3 ± 0 | 0.4 ± 0 | 0.3 ± 0 | 0.001 | 0.7 ± 0.1 | 0.4 ± 0 | 0.5 ± 0.1 | 0.4 ± 0 | <0.001 |
| **Untaxed food purchases (g/capita/day) (mean ± SE)** | | | | | | | | | | | | | | | | | | | | |
| Convenience stores | 0.6 ± 0 | 0.6 ± 0 | 0.6 ± 0 | 0.6 ± 0 | 0.942 | 0.4 ± 0.1 | 0.4 ± 0.1 | 0.4 ± 0.1 | 0.4 ± 0.1 | 0.775 | 0.5 ± 0.1 | 0.5 ± 0 | 0.5 ± 0.1 | 0.5 ± 0.1 | 0.665 | 0.9 ± 0.1 | 1.0 ± 0.1 | 1.0 ± 0.1 | 0.8 ± 0.1 | 0.548 |
| Supermarkets | 25.2 ± 0.5 | 26.3 ± 0.6 | 25.7 ± 0.6 | 23.3 ± 0.6 | 0.007 | 13.7 ± 0.7 | 15.4 ± 0.8 | 15.9 ± 0.9 | 14.8 ± 1.0 | 0.425 | 24.1 ± 0.6 | 26.5 ± 0.8 | 25.8 ± 0.8 | 23.3 ± 0.8 | 0.365 | 38.8 ± 1.2 | 36.8 ± 1.1 | 36.3 ± 1.3 | 32.5 ± 1.3 | 0.001 |
| Wholesalers/price clubs | 2.0 ± 0.1 | 2.2 ± 0.1 | 2.5 ± 0.2 | 2.1 ± 0.2 | 0.424 | 0.6 ± 0.1 | 0.6 ± 0.1 | 0.7 ± 0.2 | 0.6 ± 0.1 | 0.95 | 1.3 ± 0.1 | 1.5 ± 0.2 | 2.1 ± 0.2 | 1.8 ± 0.2 | 0.008 | 5.3 ± 0.5 | 5.1 ± 0.4 | 5.2 ± 0.4 | 4.5 ± 0.7 | 0.346 |

| | | | | | | | | | | | | | | | | | | | | |
|---|---|---|---|---|---|---|---|---|---|---|---|---|---|---|---|---|---|---|---|---|
| Traditional retailers | 17.3 ± 0.3 | 16.9 ± 0.3 | 16.4 ± 0.3 | 14.7 ± 0.3 | <0.001 | 21.0 ± 0.7 | 19.8 ± 0.7 | 18.9 ± 0.7 | 17.2 ± 0.7 | <0.001 | 17.8 ± 0.4 | 17.4 ± 0.5 | 16.9 ± 0.5 | 15 ± 0.4 | <0.001 | 12.5 ± 0.5 | 13.0 ± 0.5 | 12.5 ± 0.5 | 11.0 ± 0.6 | 0.038 |
| Others | 2.8 ± 0.1 | 2.1 ± 0.1 | 2.3 ± 0.1 | 2.0 ± 0.1 | <0.001 | 2.3 ± 0.2 | 1.6 ± 0.2 | 1.7 ± 0.2 | 1.5 ± 0.2 | 0.007 | 2.6 ± 0.1 | 1.9 ± 0.1 | 2.0 ± 0.1 | 1.9 ± 0.1 | 0.001 | 3.6 ± 0.2 | 3.1 ± 0.3 | 3.7 ± 0.5 | 2.7 ± 0.3 | 0.075 |

Source: Authors' own analyses and calculations based on data from Nielsen through its Mexico Consumer Panel Service (CPS) for the food and beverage categories for January 2012 – December 2015. The Nielsen Company, 2016. Nielsen is not responsible for and had no role in preparing the results reported herein. Multivariate linear regression models were used to predict unadjusted means of per capita daily purchases of taxed and untaxed foods according to store-type, SES and year of purchases. Means were weighted to be urban representative. Our statistical testing focused on the trends analysis.
